# Comparative Proteomic Analysis Reveals Differential Root Proteins in *Medicago sativa* and *Medicago truncatula* in Response to Salt Stress

**DOI:** 10.3389/fpls.2016.00424

**Published:** 2016-03-31

**Authors:** Ruicai Long, Mingna Li, Tiejun Zhang, Junmei Kang, Yan Sun, Lili Cong, Yanli Gao, Fengqi Liu, Qingchuan Yang

**Affiliations:** ^1^Institute of Animal Sciences, Chinese Academy of Agricultural SciencesBeijing, China; ^2^Department of Grass and Forage Science, College of Animal Science and Technology, China Agricultural UniversityBeijing, China; ^3^Institute of Pratacultural Science, Heilongjiang Academy of Agricultural SciencesHaerbin, China

**Keywords:** *Medicago*, salt stress, root, protein, 2-DE, gene expression, function

## Abstract

Salt stress is an important abiotic stress that causes decreased crop yields. Root growth and plant activities are affected by salt stress through the actions of specific genes that help roots adapt to adverse environmental conditions. For a more comprehensive understanding of proteins affected by salinity, we used two-dimensional gel electrophoresis and mass spectrometry to characterize the proteome-level changes associated with salt stress response in *Medicago sativa* cv. Zhongmu-1 and *Medicago truncatula* cv. Jemalong A17 roots. Our physiological and phenotypic observations indicated that Zhongmu-1 was more salt tolerant than Jemalong A17. We identified 93 and 30 proteins whose abundance was significantly affected by salt stress in Zhongmu-1 and Jemalong A17 roots, respectively. The tandem mass spectrometry analysis of the differentially accumulated proteins resulted in the identification of 60 and 26 proteins in Zhongmu-1 and Jemalong A17 roots, respectively. Function analyses indicated molecule binding and catalytic activity were the two primary functional categories. These proteins have known functions in various molecular processes, including defense against oxidative stress, metabolism, photosynthesis, protein synthesis and processing, and signal transduction. The transcript levels of four identified proteins were determined by quantitative reverse transcription polymerase chain reaction. Our results indicate that some of the identified proteins may play key roles in salt stress tolerance.

## Introduction

Plant growth and productivity are adversely affected by various natural abiotic and biotic factors, which cause considerable crop losses worldwide. These factors prevent plants from reaching their full genetic potential and limit crop productivity (Cramer et al., 2011). Salt stress is an important abiotic factor in many parts of the world, especially on irrigated lands (Munns and Tester, 2008). Soil salinity is a major abiotic stress influencing crop production, and researchers have investigated plant salt tolerance mechanisms with the aim of improving crop plants (Duzan et al., 2004). The metabolic imbalances caused by ion toxicity, osmotic stress, and nutritional deficiency due to salinity may also lead to oxidative stress (Zhu, 2002). These negative effects trigger changes to root morphology and suppression of plant growth, and can ultimately result in plant death.

Salinity regulates the expression of many plant genes at the transcriptional and post-translational levels. The molecular mechanism of plant salt tolerance is very complex (Zhu, 2001, 2002; Munns and Tester, 2008). To investigate this mechanism, several studies have been conducted in many plant models. Published analyses have helped characterize the expression profiles of many genes and proteins involved in salt stress responses in *Arabidopsis thaliana*, rice, wheat, soybean, tobacco, barrel medic (*Medicago truncatula*), and other plant species (Merchan et al., 2007; Cheng et al., 2009; Kumari et al., 2009; Razavizadeh et al., 2009; Zhang et al., 2009; Sobhanian et al., 2010; Capriotti et al., 2014; Ghaffari et al., 2014). The root is the primary tissue involved in salinity perception and is one of the first to be injured following exposure to several types of stresses. The sensitivity of the root to stress often limits the productivity of the entire plant (Steppuhn et al., 2010). Therefore, a comprehensive understanding of root molecular responses to salt stress is necessary for researchers to be able to increase crop tolerance to salt stress.

Plants differ considerably in their tolerance to salinity, as reflected by their different growth responses. For instance, several legumes, including *Medicago sativa* (alfalfa) and *M. truncatula*, have cultivars that have adapted to saline soils. This adaptive process is associated with a number of biochemical and physiological changes. The majority of these modifications are regulated by salt through alterations in gene expression (Zhu, 2002; Munns and Tester, 2008). Proteomics-based technologies have become powerful tools in the study of protein expression (Faurobert et al., 2007). For example, the combination of two-dimensional gel electrophoresis (2-DE) and mass spectrometry has been one of the most widely used techniques to study plant proteomes. Investigations into plant proteome changes during exposure to salt stress have been conducted for many plants, such as *A. thaliana* (Jiang et al., 2007), tomato (Manaa et al., 2011), soybean (Sobhanian et al., 2010), rice (Zhang et al., 2009; Ghaffari et al., 2014), tobacco (Razavizadeh et al., 2009), durum wheat (Capriotti et al., 2014), and barley (Witzel et al., 2014).

In this study, we explored new potential regulatory proteins of salt stress tolerance in *M. sativa* and *M. truncatula* roots. Many *M. truncatula* cultivars are more salt sensitive than *M. sativa*. Some *M. sativa* cultivars are highly tolerant to salinity stress (Munns and Tester, 2008), particularly *M. sativa* L. cv. Zhongmu-1, which we developed. High salt concentrations surrounding plant roots can induce rapid changes to cell growth and associated metabolic activities. The accumulation of salts inside plants can be toxic (Munns and Tester, 2008). There have been many genetic studies of *Medicago* species, but most of them focused on the model legume, *M. truncatula.* The objective of our study was to identify novel proteins regulated by salt stress in *M. sativa* and *M. truncatula* roots. We also aimed to determine differences in protein expression patterns between these two leguminous plants. We prepared total protein extracts from *M. sativa* and *M. truncatula* seedling roots treated with 300 mM NaCl and compared them to those of control roots using 2-DE. We identified novel salt stress-responsive root proteins and differentially expressed proteins.

## Materials and methods

### Plant materials

We used *M. sativa* cv. Zhongmu-1 and *M. truncatula* cv. Jemalong A17 in this study. Zhongmu-1 is a salt-tolerant cultivar of alfalfa (*M. sativa*, tetraploid, 2*n* = 4 × = 32), which is widely cultivated in China. Jemalong A17 is a cultivar of *M. truncatula* (diploid, 2*n* = 16), which is salt-sensitive. The genome sequence of Jemalong A17 is already known. Seeds of both cultivars were surface-sterilized in 75% ethanol for 10 min followed by three washes with sterile water. Seeds were germinated on moistened Whatman filter paper placed in Petri dishes (10 cm diameter). After a week, the seedlings were transferred to hydroponic cultures containing full-strength Hoagland's solution in a growth chamber with a 16 h/8 h light/dark photoperiod at 25°C and 65% relative humidity. The Hoagland's solution was renewed every 3 days.

### Physiological analysis

One-month-old Zhongmu-1 and Jemalong A17 seedlings were treated with Hoagland's solution supplemented with 300 mM NaCl for 0, 2, 8, 24, and 48 h. They were then analyzed for relative water content (RWC), electrolyte leakage, and proline content. The RWC was used to evaluate plant water status. Leaf RWC was calculated as RWC = (FW − DW) / (WS − DW), where FW refers to fresh weight, DW refers to dry weight, and WS refers to saturated water weight. Membrane damage was assessed by measuring electrolyte leakage. For each measurement, 10 g seedlings were added to 30 ml double deionized water in 50-ml tubes. Air was removed from the tubes using a vacuum pump until all seedlings were submerged in the water. Seedlings were maintained in the water for 4 h at 25°C.

We measured the conductivity of the bathing solution with a conductivity meter (Mettler Toledo) (as L_1_). The tubes were then incubated at 100°C for 15 min. The conductivity of the incubated solution was measured again after cooling to room temperature (as L_2_). For each sample, the relative conductivity (%) was calculated as L_1_/L_2_ × 100. Samples treated with 300 mM NaCl were harvested, frozen at −80°C and ground to a fine powder in liquid nitrogen using a mortar and pestle. We measured the free proline content using a colorimetric assay as described (Bates et al., 1973). Proline concentration was determined using a calibration curve and expressed as mg proline g^−1^ FW.

### Sample preparation and 2-DE

To identify *M. sativa* and *M. truncatula* proteins potentially involved in regulating salt tolerance, three independent replicates of 1-month-old seedling root samples were collected from Zhongmu-1 and Jemalong A17 plants treated with 300 mM NaCl for 8 h (Long et al., 2015). Untreated roots were used as controls. Root samples were ground to a fine powder in liquid nitrogen. Total protein extracts were prepared from the ground root samples using an optimized TRIzol method (Xiong et al., 2011). The final protein pellets were washed three times in 1 ml ethanol and resuspended in 1 ml lysis buffer (8 M urea, 4% w/v CHAPS, and 2% w/v DTT). Protein samples were sonicated for 10 min (4°C) and incubated at room temperature for 2 h. The protein solutions were centrifuged (40,000 × g, 40 min, 4°C) and the supernatants were collected. The protein concentrations of the supernatants were determined using the 2-D Quant kit according to the manufacturer's protocol (GE Healthcare). We diluted protein solutions with rehydration buffer [8 M urea, 2% w/v CHAPS, 1% w/v DTT, 0.5% v/v immobilized pH gradient (IPG) buffer pH 4–7, and 0.002% w/v bromophenol blue]. We then loaded 120 mg protein (in 450 μl) onto pH 4–7 IPG strips (24 cm). Isoelectric focusing (IEF) was completed using the Ettan IPGphorII system (GE Healthcare). The IEF and second dimension sodium dodecyl sulfate polyacrylamide gel electrophoresis were performed as described (Xiong et al., 2011). The IEF running conditions were as follows: 30 V for 12 h, 150 V for 250 Vh, 200 V for 300 Vh, 500 V for 250 Vh, 1000 V for 1000 Vh, 8000 V for 3 h, and 8000 V for a total of 30,000 Vh. Gel electrophoresis was performed using 12% polyacrylamide gels and the Ettan DALTsix electrophoresis gel system (GE Healthcare). The proteins were visualized using colloidal Coomassie brilliant blue G-250.

### Protein visualization and image analysis

The stained gels were scanned using a UMAX Power Look 2100XL scanner (UMAX) at a resolution of 600 dots per inch. Gel images were analyzed using ImageMaster™ 2D Platinum Version 5.0 (GE Healthcare Bio-Science). We estimated the isoelectric point (pI) of the proteins based on the relative migration of the protein spots on the IPG strips. All spot volumes were normalized as a percentage of the total volume of all spots present in the gel. We used ImageMaster™ 2D Platinum Version 5.0 to perform ANOVA. Comparisons of the mean differences were completed using Duncan's multiple range test at *P* < 0.05. The protein spots were determined to be significantly up- or down-regulated when the abundance fold change was more than 1.5 at *P* < 0.05.

### Protein identification and analysis of function

Significantly up- or down-regulated protein spots were excised from gels and destained for 2 h at room temperature using a freshly prepared wash solution consisting of 100% acetonitrile, 50 mM NH_4_CHO_3_ (50:50 v/v). Proteins were digested using a trypsin solution according to an established method (Xiong et al., 2011). Peptide mixtures were analyzed using the 4800 Plus MALDI TOF/TOF^*TM*^ Analyzer (ABI), which is a matrix-assisted laser desorption ionization time of flight (MALDI-TOF/TOF) mass spectrometer. Mass spectrometry was completed using an established method (Li et al., 2011). Proteins were identified using peak lists for searches against the NCBInr database with the Mascot search engine (http://www.matrixscience.com/). The search criteria consisted of the following: Enzyme, Trypsin; Variable modifications, Oxidation (M); Peptide tolerance, 200 ppm; MS/MS tolerance, 0.8 Da; Instrument, MALDI-TOF/TOF; and Carbamidomethyl (C) as a fixed modification for all alkylated samples. Blast2GO software was used for gene ontology and Kyoto Encyclopedia of Genes and Genomes pathway analyses of identified proteins (Conesa et al., 2005).

### Transcript analysis using quantitative reverse transcription polymerase chain reaction (qRT-PCR)

We used 1-month-old Zhongmu-1 and Jemalong A17 seedlings for transcript analyses. Total RNA was isolated from roots treated with NaCl (0, 2, 8, and 24 h) using TRIzol (Invitrogen, USA) according to the manufacturer's instructions. The RNA was then reverse transcribed and the synthesized cDNA was used as the template for qRT-PCR. The real-time fluorescent quantitative PCR was completed using the ABI 7500 system (Applied Biosystems). The β*-actin* gene served as a housekeeping gene to normalize target gene quantities. The real-time PCR primers used for the amplification of β*-actin* and the genes of ten identified proteins are listed in Supplementary Table 1. The PCR program consisted of a maximum of 40 cycles of 95°C for 15 s and 60°C for 30 s, followed by melting curve analysis. Transcript abundance for each gene was normalized to that of β*-actin*. The relative expression levels were calculated as follows: ratio = 2^−ΔΔCt^ = 2^−[Ct, t−Ct, r]^, where Ct refers to cycle threshold, Ct,t refers to Ct of the target gene and Ct,r refers to Ct of the β*-actin* control gene.

## Statistical analysis

All experiments were repeated with three independent biological replicates. All data obtained were subjected to a one-way ANOVA. The mean differences were compared using Duncan's multiple range *t*-test. Comparisons with *P* < 0.05 were considered significantly different. The values provided in the figures and tables are the means ± standard errors.

## Results

### Physiological parameters related to salt tolerance

**Figure 1:**
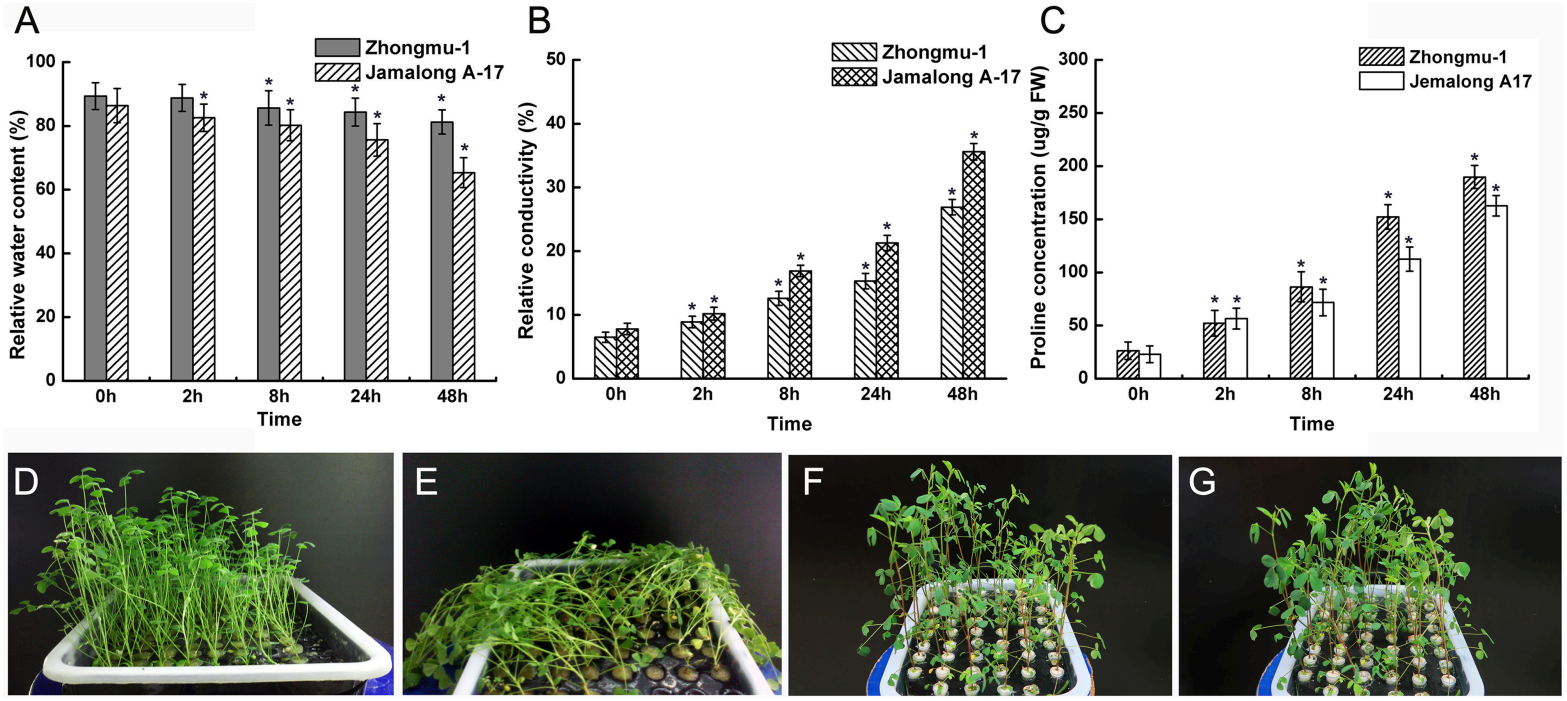
**Physiological analysis and phenotypic observation of *M. sativa* cv. Zhongmu-1 and *M. truncatula* cv. Jemalong A17 under salt stress**. One-month-old Zhongmu-1 and Jemalong A17 seedlings treated with 300 mM NaCl for 0, 2, 8, 24, and 48 h were analyzed for relative water content **(A)**, electrolyte leakage **(B)**, and proline content **(C)**. **(D,E)** phenotypic observation of 1-month-old Jemalong A17 seedlings treated with 300 mM NaCl for 0 and 8 h. **(F,G)** phenotypic observation 1-month-old Zhongmu-1 seedlings treated with 300 mM NaCl for 0 and 8 h. ^*^ Indicates significant difference at *p* < 0.05 (Student's *t*-test).

The responses of Zhongmu-1 and Jemalong A17 to salt stress were compared in terms of leaf RWC, electrolyte leakage, and proline content. We observed that the leaf RWC of Zhongmu-1 and Jemalong A17 decreased by about 10% and over 20%, respectively, after exposure to salt stress for 48 h (Figure 1A). Relative electrolyte conductivity and proline content increased dramatically in salt stressed plants (Figure 1B). Following salt treatment, the relative electrolyte conductivity of Zhongmu-1 was lower than that of Jemalong A17. Conversely, proline accumulation in Zhongmu-1 was higher than that of Jemalong A17 (Figure 1C). Acording to the phenotypic observation the wilting degree of Jemalong A17 seedlings was much more obvious than that of Zhongmu-1 seedlings after treating with 300 mM NaCl for 8 h (Figures 1D–G). The physiological and phenotypic observations confirmed that *M. sativa* cv. Zhongmu-1 is more salt-tolerant than *M. truncatula* cv. Jemalong A17.

### Protein responses to salt stress

**Figure 2:**
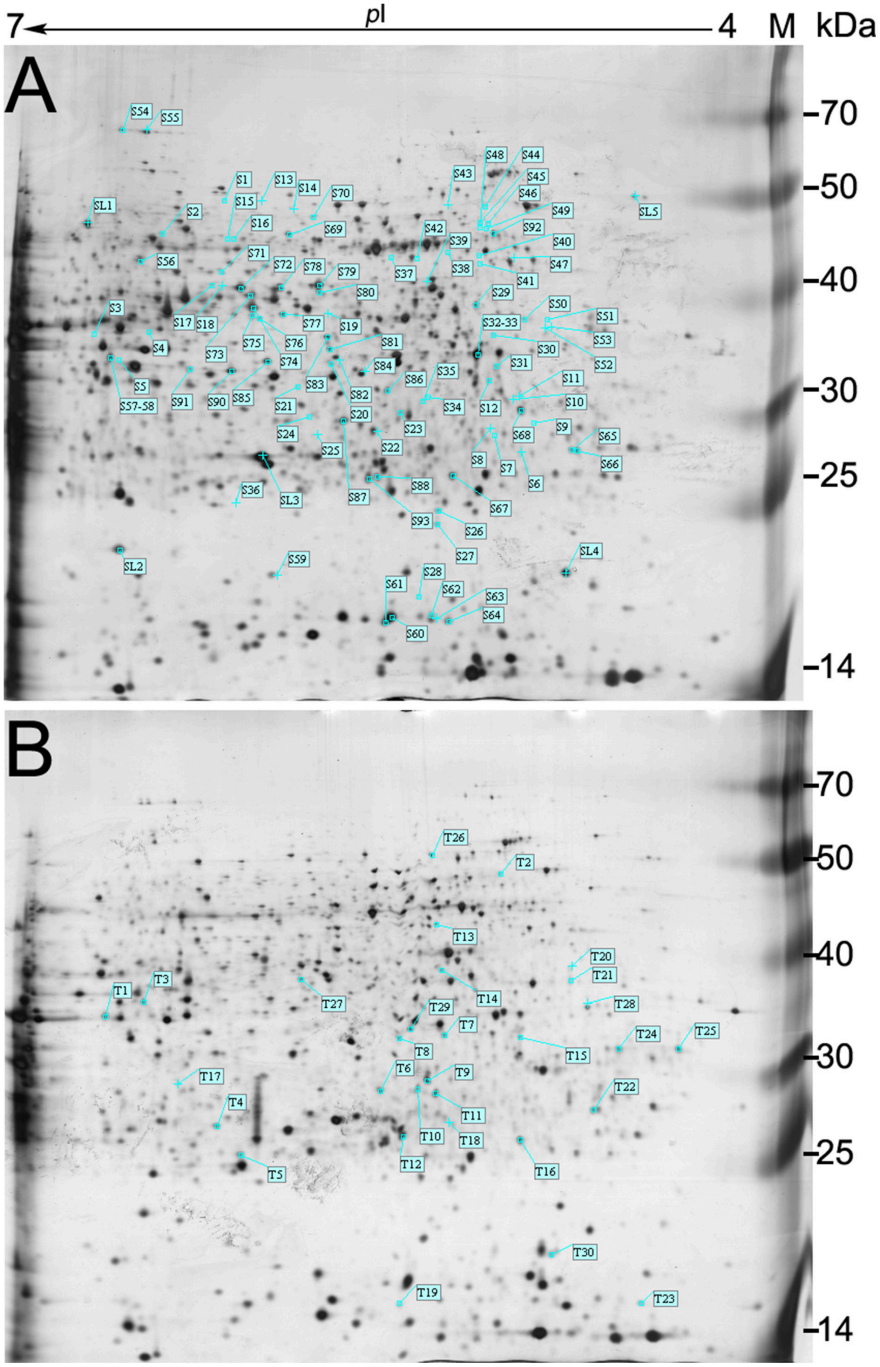
**Representative 2-DE gel images of *M. sativa* cv. Zhongmu-1 and *M. truncatula* cv. Jemalong A17 root proteins**. There were 93 and 30 protein spots in Zhongmu-1 **(A)** and Jemalong A17 **(B)**, respectively, showing at least a 1.5-fold change following 300 mM NaCl treatment (*P* < 0.05). M, protein marker.

**Figure 3:**
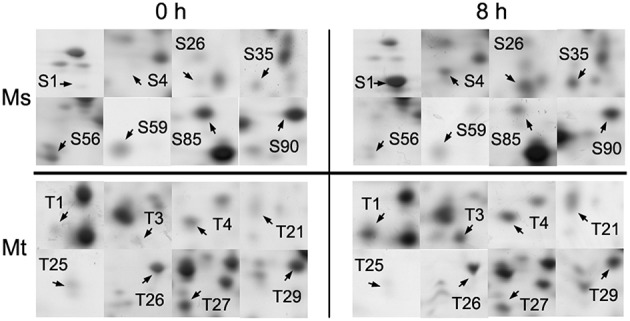
**Enlarged 2-DE gel regions of 16 differentially accumulated root proteins in *M. sativa* cv. Zhongmu-1 (Ms) and *M. truncatula* cv. Jemalong A17 (Mt)**. The abundance of S1, S4, S26, S35, T1, T3, T4, and T21 increased after 8 h salt stress. The abundance of S56, S59, S85, S90, T25, T26, T27, and T29 decreased after 8 h salt stress.

The root is the first plant organ to be affected by salt stress. Representative 2-DE gel images of the Zhongmu-1 and Jemalong A17 root proteomes following salt treatment are presented in Figure 2. There was a broad distribution of the proteins in terms of pI (4.0–7.0) and mass (10–70 kDa). Of the approximately 800 detected Zhongmu-1 protein spots, 93 exhibited significant changes to spot abundance (*P* < 0.05) (Figure 2A, Supplementary Figure 1). Fifty-three of these proteins were up-regulated by salt stress, with the remaining 40 being down-regulated (Supplementary Table 2, Figure 3). Of the approximately 900 detected Jemalong A17 protein spots, 30 protein spots exhibited significant changes to spot abundance (*P* < 0.05) (Figure 2B, Supplementary Figure 1). Twenty-two of these proteins were up-regulated by salt stress, with the remaining eight being down-regulated (Supplementary Table 2, Figure 3).

### Protein identification

After mass spectrometric analysis, a total of 60 and 26 protein spots were identified in Zhongmu-1 and Jemalong A17, respectively (Table 1, S and T correspond to protein spots of Zhongmu-1 and Jemalong A17). The mass spectrometry proteomics data have been deposited to the ProteomeXchange Consortium via the PRIDE (Vizcaino et al., 2014) partner repository with the dataset identifier PXD003761. These proteins were classified into five groups according to their molecular function as follows: Anti-oxidation, photosynthesis, metabolism, signal transduction, and protein synthesis and processing. Only the following proteins were identified in Zhongmu-1 and Jemalong A17: chaperonin CPN60-like protein (S44 and T2), fructose-bisphosphate aldolase (S3 and T1), and heat shock protein (S28 and T26). The abundance of chaperonin CPN60-like protein and fructose-bisphosphate aldolase increased in Zhongmu-1 and Jemalong A17 following salt treatment. However, the response of the heat shock protein corresponding to S28 and T26 differed between Zhongmu-1 and Jemalong A17. We observed an increase in S28 abundance and a decrease in T26 following salt treatment. Some proteins were identified in more than one spot on the same gel (Table 1). For example, S3 and S4 were identified as fructose-bisphosphate aldolase, S17 and S53 were identified as phosphopyruvate hydratase, S54 and S55 were identified as aconitate hydratase, and S61, S62, S63, and S64 were identified as a translation initiation factor (eIF-5A). Further examination of electrophoresis patterns indicated that the inferred mass and pI values of these spots differed, perhaps because of post-translational modifications or degradations.

**Table 1:** **Identities of salt-responsive proteins in *M. sativa* cv. Zhongmu-1 and *M. truncatula* cv. Jemalong A17 based on mass spectrometry and a Mascot search**.

**Spot ID**	**Genbank ID**	**Putative identity**	**Species^†^**	**pI/MW(kDa)**	**Score^††^**	**Fold change^†††^**
S1	gi|3914590	Ribulose bisphosphate carboxylase	*G. tomentella*	8.87/20.1	89	47.46 ± 2.51
S2	gi|7240134	Ribulose-1,5-bisphosphate carboxylase/oxygenase large subunit	*B. sinensis*	6.32 /52.2	224	7.47 ± 0.86
S3	gi|357490465	Fructose-bisphosphate aldolase	*M. truncatula*	5.76/78.7	780	40.62 ± 3.26
S4	gi|357490465	Fructose-bisphosphate aldolase	*M. truncatula*	5.76/78.7	162	18.74 ± 1.58
S6	gi|224072140	Chromosome segregation ATPase	*P. trichocarpa*	4.78/14.7	84	32.06 ± 2.57
S7	gi|168067236	unknown	*P. patens*	9.77/47.3	81	75.88 ± 5.36
S9	gi|217071356	Plasma membrane-associated cation-binding protein 1-like	*M. truncatula*	4.93/23.7	168	39.76 ± 1.23
S12	gi|168038256	unknown	*P. patens*	9.09/20.4	79	4.08 ± 0.27
S17	gi|388514639	phosphopyruvate hydratase	*M. truncatula*	5.55/20.3	206	2.56 ± 0.35
S18	gi|160895838	Elongation factor Tu	*D. acidovorans*	5.48/43.2	82	69.93 ± 4.56
S20	gi|223943929	DUF827 domain containing family protein	*Z. mays*	5.2/87.4	76	2.33 ± 0.35
S22	gi|357475283	Caffeoyl-CoA O-methyltransferase	*M. truncatula*	5.42/28.1	119	199.75 ± 8.95
S24	gi|255083855	photosystem II PsbR protein	*Micromonas sp. RCC299*	9.9/13.3	60	35.21 ± 5.36
S26	gi|388506824	Lipid transfer protein	*M. truncatula*	5.58/23.8	263	3.93 ± 0.56
S28	gi|357476131	Heat shock protein	*M. truncatula*	5.87/72.4	124	72.44 ± 5.12
S29	gi|44887779	Methyltransferase	*M. sativa*	5.1/41.5	141	3.19 ± 0.31
S30	gi|255085468	Unknown	*Micromonas sp. RCC299*	6.13/16.5	80	5.88 ± 0.52
S32	gi|357454485	pfkB family carbohydrate kinase	*M. truncatula*	5.2/35.3	647	1.61 ± 0.17
S35	gi|255623263	CD4+ T-cell-stimulating antigen precursor	*R. communis*	9.26/22.9	84	54.20 ± 8.59
S37	gi|115531966	Chloroplast RF1	*P. x hortorum*	10.05/30.9	76	45.64 ± 4.56
S40	gi|302755124	ATP synthase subunit beta	*S. moellendorffii*	5.2/46.5	172	33.36 ± 2.59
S42	gi|222870503	ATP synthase subunit beta	*D. acidovorans*	5.04/34.1	140	26.94 ± 4.26
S44	gi|222872490	TCP-1/cpn60 chaperonin family protein	*M. truncatula*	5.06/36.1	230	9.92 ± 0.56
S47	gi|170294005	ATP synthase beta subunit	*Cladophora sp. CHR505640*	5.08/39.6	160	35.37 ± 2.51
S48	gi|18402291	AT hook motif-containing protein	*A. thaliana*	11.42/21.6	76	68.81 ± 4.23
S53	gi|388514649	phosphopyruvate hydratase	*M. truncatula*	5.08/38.1	528	199.51 ± 15.89
S54	gi|357483921	Aconitate hydratase	*M. truncatula*	6.1/98.8	668	0.19 ± 0.03
S55	gi|357453423	Aconitate hydratase	*M. truncatula*	7.6/107.5	969	0.18 ± 0.05
S56	gi|388513787	Monodehydroascorbate reductase	*M. truncatula*	7.03/36.2	206	0.25 ± 0.04
S57	gi|357507415	Cysteine synthase	*M. truncatula*	8.28/41.1	562	0.54 ± 0.10
S59	gi|357467311	Universal stress protein A-like protein	*M. truncatula*	5.97/19.8	638	0.63 ± 0.03
S60	gi|388504476	Dehydrase and lipid transporter	*M. truncatula*	5.31/18.1	577	0.61 ± 0.08
S61	gi|20138664	Eukaryotic translation initiation factor	*M. sativa*	5.41/17.5	340	0.54 ± 0.06
S62	gi|115473151	Translation initiation factor, eIF-5A	*O. sativa*	5.77/17.6	249	0.09 ± 0.02
S63	gi|8919176	Translation initiation factor, eIF-5A	*O. sativa*	5.71/17.7	329	0.02 ± 0.01
S64	gi|89276313	Eukaryotic translation initiation factor 5A	*G. conopsea*	5.88/17.5	465	0.04 ± 0.01
S65	gi|297737737	ATP synthase subunit beta	*V. vinifera*	5.27/45.3	134	0.46 ± 0.02
S66	gi|357444609	ATP synthase subunit beta	*M. truncatula*	5.86/12.1	817	0.61 ± 0.06
S67	gi|388516225	Glutathione S-transferase tau	*M. truncatula*	5.58/27.1	443	0.34 ± 0.02
S69	gi|356528649	Inosine-5′-monophosphate dehydrogenase	*G. max*	8.58/43.1	282	0.73 ± 0.07
S70	gi|357513617	Chaperonin CPN60-like protein	*M. truncatula*	7.03/61.9	177	0.29 ± 0.05
S71	gi|84468322	DNA binding protein	*T. pratense*	6.11/44.2	720	0.22 ± 0.06
S72	gi|223635319	S-adenosyl-L-methionine synthase	*M. falcata*	5.77/43.5	952	0.62 ± 0.04
S73	gi|537317	Peroxidase	*M. sativa*	5.76/38.7	397	0.20 ± 0.02
S75	gi|357451633	Phosphoglycerate kinase	*M. truncatula*	5.61/42.6	329	-
S76	gi|388493272	Cinnamyl alcohol dehydrogenase-like protein	*M. truncatula*	6.42/39.5	98	-
S77	gi|261889456	S-adenosyl-L-methionine synthase	*M. sativa*	5.67/40.3	653	0.59 ± 0.06
S79	gi|729442	Probable protein disulfide-isomerase	*M. sativa*	5.44/40.8	856	0.49 ± 0.08
S80	gi|357499179	Dihydropyrimidine dehydrogenase	*M. truncatula*	7.05/46.8	237	-
S81	gi|225441423	Enoyl-[acyl-carrier-protein] reductase	*V. vinifera*	8.92/41.6	428	0.50 ± 0.09
S82	gi|388491488	Quinone oxidoreductase-like protein	*M. truncatula*	5.53/33.8	937	0.51 ± 0.07
S83	gi|357512299	Aldo/keto-reductase family protein	*M. truncatula*	6.13/38.6	517	0.44 ± 0.03
S84	gi|512400	Annexin	*M. sativa*	5.41/34.9	861	0.60 ± 0.05
S85	gi|388500780	Fructose-bisphosphate aldolase	*L. japonicus*	8.22/42.5	456	0.56 ± 0.03
S86	gi|388505104	Thylakoid-bound ascorbate peroxidase	*M. truncatula*	8.73/40.4	725	0.49 ± 0.02
S87	gi|357501457	Epsin-2	*M. truncatula*	7.55/91.3	380	0.43 ± 0.02
S88	gi|357444971	Chalcone-flavonone isomerase 2-B	*M. truncatula*	5.63/25.1	140	0.41 ± 0.01
S90	gi|2827084	Malate dehydrogenase precursor	*M. sativa*	8.11/43.4	1310	0.53 ± 0.06
S91	gi|357465811	Omega-amidase NIT2	*M. truncatula*	7.11/39.3	463	-
S93	gi|388511263	GroES chaperonin	*M. truncatula*	9.02/27.1	565	0.56 ± 0.08
T1	gi|357490465	Fructose-bisphosphate aldolase	*M. truncatula*	5.76/78.7	557	19.87 ± 1.25
T2	gi|222872490	60 kDa chaperonin	*D. acidovorans*	5.06/36.1	233	30.16 ± 3.26
T4	gi|388495516	Glyceraldehyde-3-phosphate dehydrogenase	*M. truncatula*	6.25/36.6	651	1.73 ± 0.15
T5	gi|388503392	Proteasome subunit alpha type	*M. truncatula*	5.92/27.5	864	1.57 ± 0.11
T6	gi|255640620	Proteasome subunit beta type	*G. max*	6.88/22.8	320	1.54 ± 0.03
T7	gi|357473913	Enoyl-ACP reductase	*M. truncatula*	9.01/39.4	460	1.56 ± 0.08
T8	gi|388500360	CHP-rich zinc finger protein	*M. truncatula*	5.97/21.0	566	1.99 ± 0.07
T9	gi|357438145	Cysteine proteinase	*M. truncatula*	6.55/40.5	479	1.58 ± 0.14
T10	gi|357490351	Hydroxyacylglutathione hydrolase	*M. truncatula*	5.36/28.7	582	2.11 ± 0.06
T11	gi|363541981	Caffeoyl-CoA 3-o-methyltransferase	*M. sativa*	5.4/26.6	569	1.63 ± 0.05
T12	gi|388507284	Triosephosphate isomerase	*M. truncatula*	6.54/33.6	449	1.56 ± 0.09
T13	gi|357463609	Argininosuccinate synthase	*M. truncatula*	6.26/53.0	609	2.40 ± 0.07
T15	gi|357440891	Polyadenylate-binding protein	*M. truncatula*	4.57/21.0	514	1.60 ± 0.05
T16	gi|217072458	L-ascorbate peroxidase	*M. truncatula*	5.52/27.1	457	4.10 ± 0.79
T17	gi|357444609	ATP synthase subunit beta	*M. truncatula*	5.86/121.2	720	1.67 ± 0.15
T18	gi|357483543	Soluble inorganic pyrophosphatase	*M. truncatula*	5.52/24.3	420	3.67 ± 0.63
T19	gi|357500689	Peroxiredoxin	*M. truncatula*	5.59/17.5	617	3.82 ± 0.45
T20	gi|388495024	Peroxidase	*M. truncatula*	4.85/37.8	200	2.07 ± 0.15
T22	gi|288816173	Oxygen-evolving enhancer protein	*T. aurea*	4.78/14.2	83	2.86 ± 0.25
T23	gi|357505041	3-demethylubiquinone-9 3-methyltransferase domain protein	*M. truncatula*	4.68/17.1	571	0.51 ± 0.05
T24	gi|356576095	Ran-binding protein 1 homolog b-like	*G. max*	4.78/24.9	336	0.53 ± 0.06
T26	gi|357476131	Heat shock protein	*M. truncatula*	5.87/72.4	860	0.62 ± 0.04
T27	gi|388502338	Caffeic acid O-methyltransferase	*M. truncatula*	5.67/40.3	799	0.60 ± 0.03
T28	gi|357493805	Desiccation protectant protein Lea14-like protein	*M. truncatula*	4.8/36.2	394	0.44 ± 0.09
T29	gi|388499976	Annexin	*M. truncatula*	5.45/28.6	611	0.53 ± 0.07
T30	gi|388508178	PITH domain plant protein	*M. truncatula*	4.99/19.9	568	0.56 ± 0.03

† Species of the matched protein based on a Mascot search.

†† Mascot search score.

††† Spot volume fold change corresponding to spot volume after 8 h salt treatment/spot volume before salt treatment (0 h).

### Functional analysis of identified proteins

**Figure 4:**
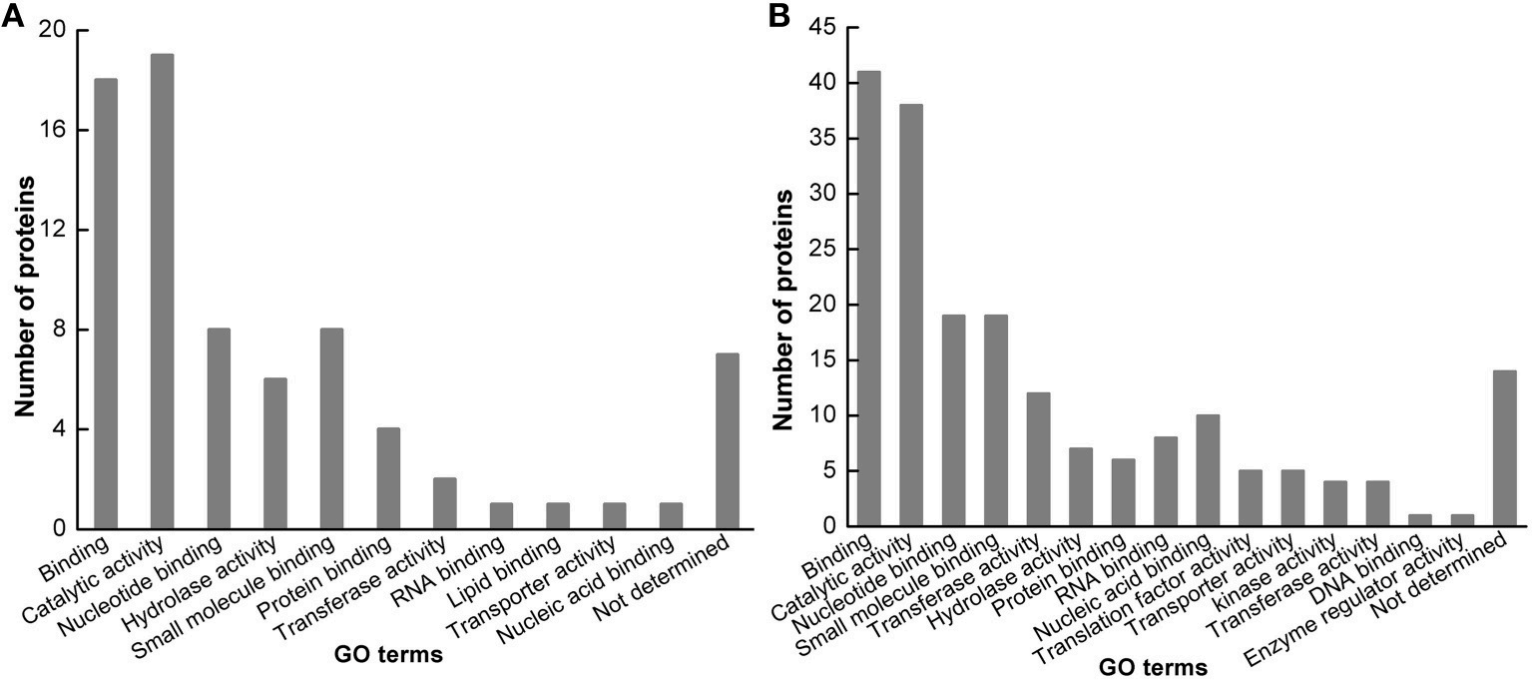
**Functional categorization of identified proteins**. The identified proteins in *M. truncatula* cv. Jemalong A17 **(A)** and *M. sativa* cv. Zhongmu-1 **(B)** were grouped into 12 and 16 functional categories, respectively.

The identified protein sequences were blasted by BLASTP in the NCBI database. These identified proteins in Jemalong A17 were classified into 11 functional groups based on GO prediction (Figure 4A), including binding, catalytic activity, nucleotide binding, hydrolase activity, small molecule binding, protein binding, transferase activity, RNA binding, lipid binding, transporter activity, nucleic acid binding and not determined. These identified proteins in Zhongmu-1 were classified into 15 functional groups based on GO prediction (Figure 4B), including binding, catalytic activity, nucleotide binding, small molecule binding, transferase activity, hydrolase activity, protein binding, RNA binding, nucleic acid binding, translation factor activity, transporter activity, kinase activity, transferase activity, DNA binding, enzyme regulator activity and not determined. Molecule binding group and catalytic activity group were the two mainly functional groups.

### Transcript analysis of selected proteins

**Figure 5:**
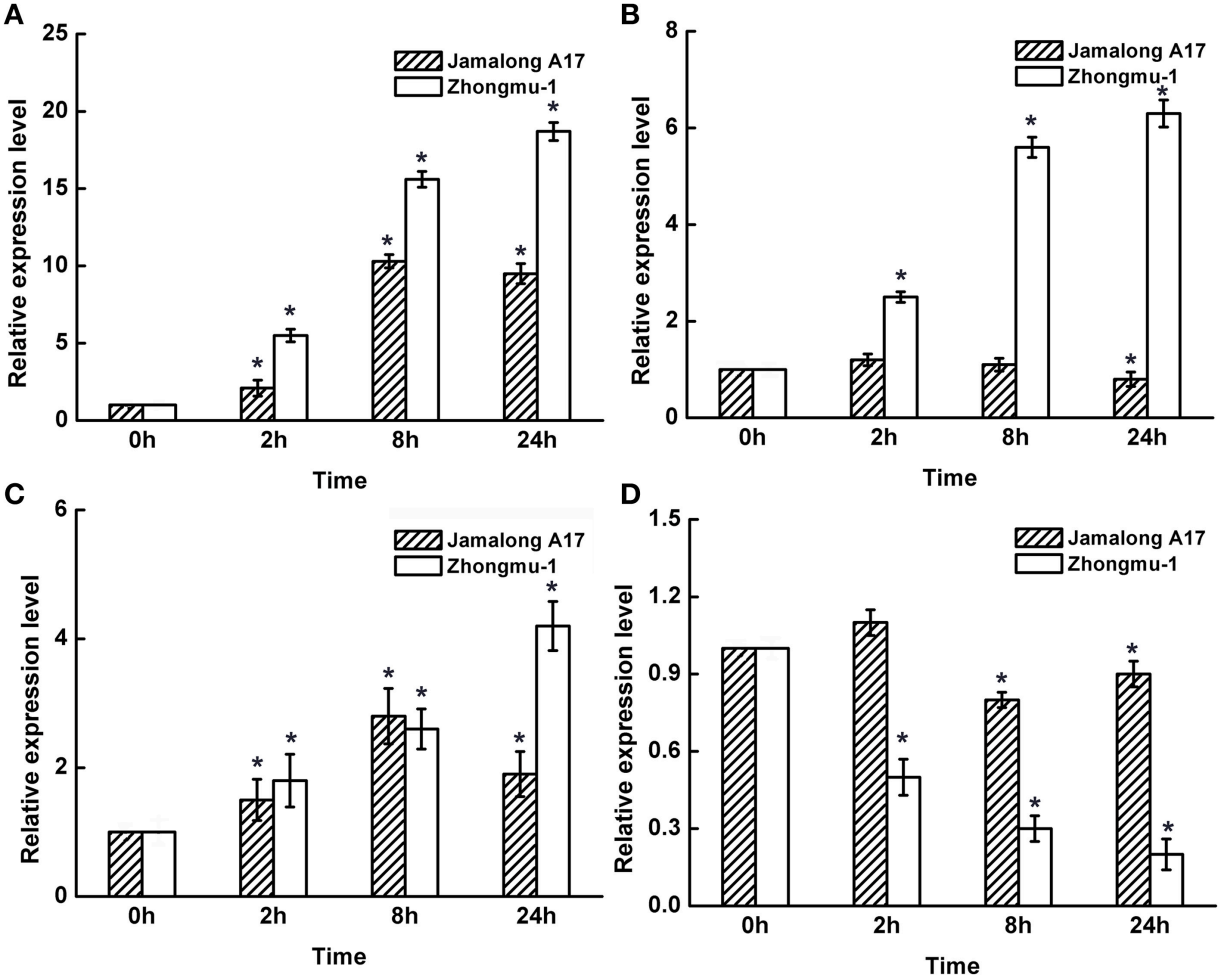
**Transcript expression levels of fructose-bisphosphate aldolase (A), heat shock protein (B), TCP-1/cpn60 chaperonin family protein (C), and cinnamyl alcohol dehydrogenase-like protein (D) in *M. sativa* cv. Zhongmu-1 and *M. truncatula* cv. Jemalong A17 roots treated with NaCl**. ^*^ Indicates significant difference at *p* < 0.05 (Student's *t*-test).

Ten differentially accumulated root proteins identified from Zhongmu-1 and Jemalong A17 were chosed to perform transcript expression analyses. The expression levels of fructose-bisphosphate aldolase (S3/T1), heat shock protein (S28/T26), TCP-1/cpn60 chaperonin family protein (S44/T2), and cinnamyl alcohol dehydrogenase-like protein (S76) based on qRT-PCR analyses are provided in Figure 4. The expression analyses results of all the 10 genes are provided in Supplementary Table 3. Compared with the expression level at 0 h, transcript abundance of fructose-bisphosphate aldolase, heat shock protein, and TCP-1/cpn60 chaperonin family protein increased considerably after salt treatment in Zhongmu-1. The transcript abundance of cinnamyl alcohol dehydrogenase-like protein decreased significantly (*P* < 0.05) after salt treatment in Zhongmu-1, whereas that of Jemalong A-17 did not significantly change, though there was a decreasing trend (Figure 5). After salt treatment, the heat shock protein transcript abundance increased more than 6-fold in Zhongmu-1, whereas that of Jemalong A-17 showed no significant changes, but did exhibit an increasing trend (Figure 5). These results along with those from the 2-DE analyses suggest that the transcript and protein level changes of most analyzed proteins were similar.

## Discussion

The leaf RWC is one of the factors used to determine the extent of wilting after certain abiotic stresses. We observed that after salt treatment, the RWC of Zhongmu-1 was significantly higher than that of Jemalong A17, indicating Jemalong A17 was more wilted. Proline is an organic solute that helps regulate cellular osmolarity during plant responses to osmotic stress. Proline accumulation has been used as a drought-tolerance selection criterion related to membrane integrity in different plant species (Misra and Gupta, 2005). After salt treatment, Zhongmu-1 accumulated more proline than Jemalong A17. An increased rate of electrolyte leakage has been used as an indicator of cell membrane physical damage during exposure to abiotic stresses (Thiaw and Hall, 2004). Electrolyte leakage in Zhongmu-1 was lower than that of Jemalong A17, indicating that the cell membranes of Jemalong A17 were more damaged than those of Zhongmu-1. The physiological characteristics of Zhongmu-1 and Jemalong A17 were consistent with their salt tolerance levels. Based on their phenotypes, Zhongmu-1 was much more tolerant than Jemalong A17. The number of Zhongmu-1 protein spots exhibiting significant changes in abundance in response to salt stress was 3-fold higher than that of Jemalong A17. This result may be related to the fact that Zhongmu-1 is considerably more salt tolerant than Jemalong A17.

Some of the identified salt stress-regulated proteins were also reported in other plant species. For example, heat shock proteins, fructose-bisphosphate aldolase, peroxidase, DNA/RNA binding protein, and caffeoyl-CoA O-methyltransferase were detected in tomato roots after exposure to salt stress (Jiang et al., 2007; Manaa et al., 2011; Witzel et al., 2014). To evaluate the correlation between mRNA and the corresponding protein levels, the expression of 10 proteins with significant salt-induced changes in protein spot abundance was quantified by qRT-PCR. The qRT-PCR and 2-DE results suggest that the mRNA and protein level changes exhibit similar trends. These results also support the concept that post-transcriptional regulation plays an important role in stress-responsive gene expression, and indicates the importance of a combined transcriptomic and proteomic analyses (Mooney et al., 2006; Jiang et al., 2007).

We observed only a few salt stress-regulated proteins that were common between Zhongmu-1 and Jemalong A17 and there were differences in the expression patterns of these proteins. For intance, heat shock protein (70 kDa) was up-regulated in Zhongmu-1 (S28), but down-regulated in Jemalong A17 (T26). Heat shock proteins play important roles in a variety of cellular processes. They maintain proteins in their functional state and are also involved in protein translocations to subcellular compartments (Goswami et al., 2010). Heat shock proteins were previously reported to be up-regulated in tomato following exposure to cold stress (Page et al., 2010) and salt stress (Manaa et al., 2011).

Some proteins were identified in more than one spot in the same gel, such as fructose-bisphosphate aldolase (S3, S4, and S85) and phosphopyruvate hydratase (S17 and S53). Glycosylation, phosphorylation, and other post-translational modifications, which can alter the molecular weight and/or charge of proteins, may be responsible for these results. It is also possible that proteins were identified from multiple spots because of translation of alternatively spliced mRNAs (Yoshimura et al., 1999; Ndimba et al., 2005; Jiang et al., 2007). Proteomic studies have also shown that some proteins may be degraded during exposure to abiotic stress. For example, 19 different ribulose-1,5-bisphosphate carboxylase/oxygenase (Rubisco) large subunit fragments were detected in salt-treated rice roots (Yan et al., 2006). Similar phenomena have been reported in tomato root proteomes affected by salt stress (Manaa et al., 2011). The multiple fragments may also be the result of protein degradation by reactive oxygen species (ROS) during stress responses (Kingston-Smith and Foyer, 2000). Excess ROS can seriously disrupt normal plant metabolism through oxidative damage to lipids, proteins, and nucleic acids (Apel and Hirt, 2004; Askari et al., 2006; Bhushan et al., 2007). Anti-oxidative enzyme peroxiredoxin (T19) was detected in our study, which has been observed in responses to various abiotic stresses, including cold (Sarhadi et al., 2010), salinity (Ghaffari et al., 2014), and drought (Ali and Komatsu, 2006).

The proteins identified in this study are involved in various molecular processes. According to our results, some proteins associated with photosynthesis and metabolism were differentially expressed in Zhongmu-1 and Jemalong A17 following salt treatment. Ribulose-1,5-bisphosphate carboxylase/oxygenase (S1 and S2) is the most prevalent plant enzyme. It forms approximately 30-50% of the total soluble protein content in chloroplasts. The small subunit of Rubisco may be degraded because of oxidative stress (Sobhanian et al., 2010). Subsequently, the production of the large subunit may be inhibited. In our study, the abundance of the small (S1) and large (S2) subunits of Rubisco increased significantly in Zhongmu-1 8 h after salt treatment. This increase in abundance indicates that *M. sativa* can survive and photosynthesize even during moderate levels of salt stress. The increased activity of Rubisco subunits in tobacco and rice under salt stress has also been demonstrated (Kim et al., 2005; Razavizadeh et al., 2009). Therefore, it is possible that the accumulation of Rubisco in *M. sativa* reflects the increase in photorespiration during exposure to salt stress. Our results showed that the abundances of some proteins associated with energy production or transport, such as cytosolic malate dehydrogenase (S90) and glyceraldehyde-3-phosphate dehydrogenase (T2), were affected by salt in Zhongmu-1 and Jemalong A-17. Cytosolic malate dehydrogenase was reported to be responsive to salinity stress in *A. thaliana* roots (Jiang et al., 2007). However, the function of some identified proteins (such as S7, S12, and S30) are still unknown. Further research is needed to determine the functions of these proteins.

## Conclusion

Our physiological and phenotypic observations confirmed that *M. sativa* cv. Zhongmu-1 is considerably more salt tolerant than *M. truncatula* cv. Jemalong A17. We used 2-DE to explore the changes in the root proteomes of these leguminous plants as a result of exposure to salt stress. Differentially accumulated proteins identified in Zhongmu-1 and Jemalong A17 were determined to be involved in various molecular processes, most of which belonged to molecule binding and catalytic activity. Some of the identified proteins were validated or predicted to play critical roles in salt stress regulation. The identification of salt-responsive proteins provides new insights into salt stress responses and the basis for further studies to improve the salt tolerance of alfalfa and other plants.

## Author contributions

RL and QY Conceived and designed the experiments, RL, ML, and TZ performed the experiments, JK, YS, LC, YG, FL contributed to data analysis, RL, ML, and QY wrote the manuscript.

### Conflict of interest statement

The authors declare that the research was conducted in the absence of any commercial or financial relationships that could be construed as a potential conflict of interest. The reviewer, PK, and handling Editor declared their shared affiliation, and the handling Editor states that the process nevertheless met the standards of a fair and objective review.

## Supplementary material

Supplementary Figure 1 **The 2-DE images of 3 biological replicates for control and the treated samples**.

Supplementary Table 1 **Primers used for qRT-PCR to analyze transcript abundance of four proteins identified during 2-DE and mass spectrometry**.

Supplementary Table 2 **Protein spot volume fold changes of all proteins identified in Zhongmu-1 and Jemalong A17**.

Supplementary Table 3 **Transcript expression analyses results of 10 differentially accumulated root proteins in Zhongmu-1 and Jemalong A17**.
