# Supplementary figures and images for: Comparative Proteomic Analysis Reveals Differential Root Proteins in Medicago sativa and Medicago truncatula in Response to Salt Stress
Source: Front Plant Sci. 2016 Mar 31;7:424. doi: 10.3389/fpls.2016.00424 (PMC4814493; doi:10.3389/fpls.2016.00424)

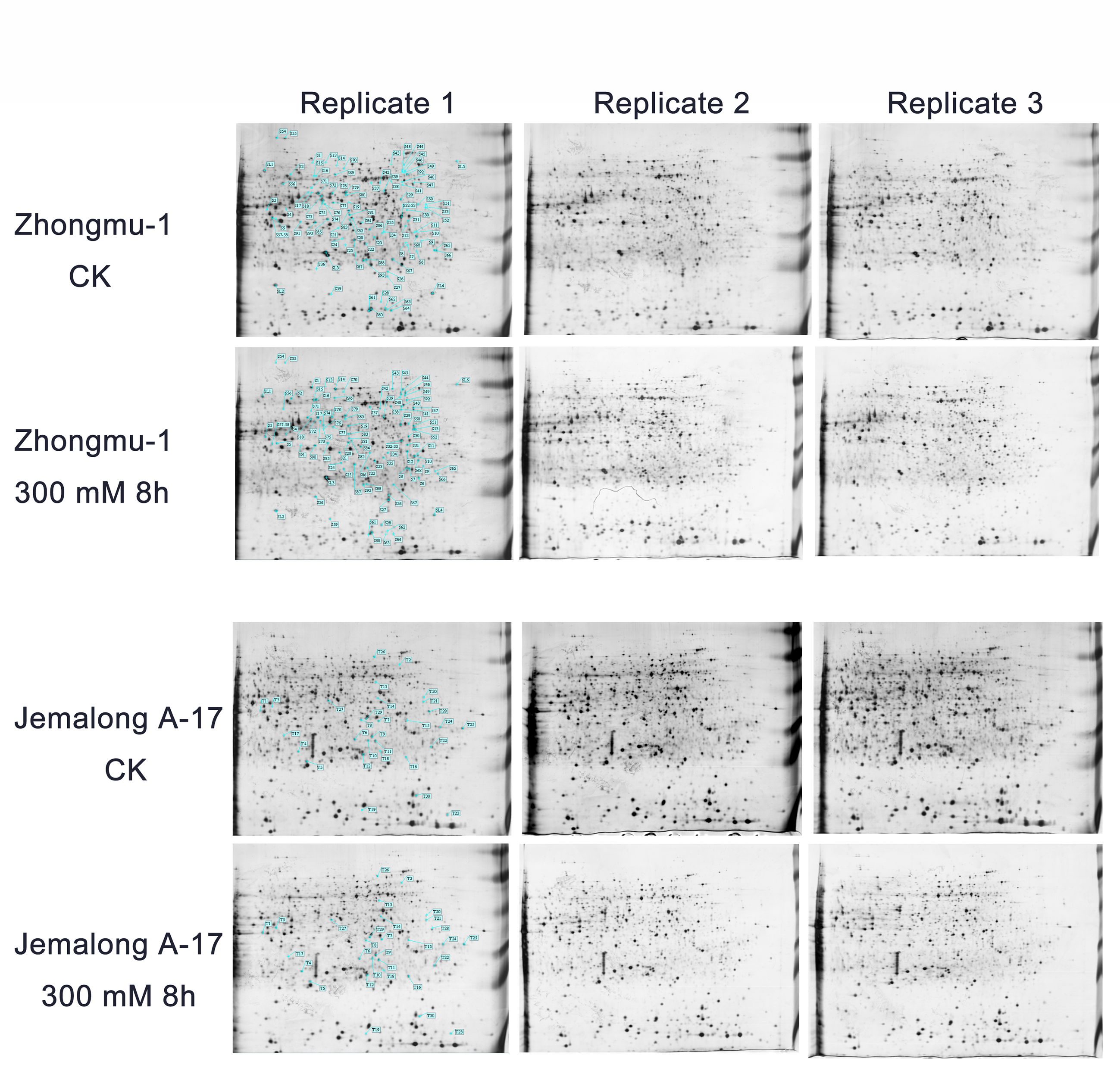

Supplement: Supplementary Figure 1 — The 2-DE images of 3 biological replicates for control and the treated samples. [file Image1.TIF]
