# Supplementary material for: Comparative Proteomic Analysis Reveals Differential Root Proteins in Medicago sativa and Medicago truncatula in Response to Salt Stress
Source: Front Plant Sci. 2016 Mar 31;7:424. doi: 10.3389/fpls.2016.00424 (PMC4814493; doi:10.3389/fpls.2016.00424)
Supplement: Supplementary Table 1 — Primers used for qRT-PCR to analyze transcript abundance of four proteins identified during 2-DE and mass spectrometry. [file Table1.DOC]

| **Spot ID/gene** | **Forward Primer (**5'-3') | | **Reverse Primer (**5'-3') |
| --- | --- | --- | --- |
| β-Actin | TGGGCTGCCACAGAACATTTGA | TGGGCTGCCACAGAACATTTGA | |
| Fructose-bisphosphate aldolase (S3/T1) | ATCCACGAGAATGCCTATGGTTTG | TCCAGGGGTAACCATGTTAGGCT | |
| Heat shock protein (S28/T26) | AGGTTCTCCAGGTGGTGGTGAT | GCTTCCAGCAATTATGTGATGATTA | |
| TCP-1/cpn60 chaperonin family protein (S44/T2) | GCCGTAATGTGGTGATTGAGC | CCAGCGACATCATTGGTAGCAT | |
| Cinnamyl alcohol dehydrogenase-like protein (S76) | AGAAGGGAAACTGGTGAAAAAGAT | CAGGAACAAGTGGATAGAAGGAAG | |
| Caffeoyl-CoA O-methyltransferase (S22/T10) | AACCCTCTTCCTACCTCTTCTCTAA | CTTCGTTGGTTGCCATTGTT | |
| Phosphopyruvate hydratase (S53) | CGGAAAGGACAAGTTTGGTGAG | TGATAAGTTGGCGATAAGGGAC | |
| S-adenosyl-L-methionine synthase (S77) | CTTTACGGTTTGGCTACTGTTGCT | GGAATGCCCCCATCAAGGACTG | |
| CHP-rich zinc finger protein (T8) | AAGAAGCCATAAACATTGCCAC | CTTTGAGCGGTTTAGCGAGAT | |
| Peroxidase (T20) | AGTTGAAAATGAATGTCCAGCC | GATCCAATGTGAAAAAGGGTGC | |
| Caffeic acid O-methyltransferase (T27) | TCCTGTCTTGGGAAGGTTTACGCT | TCAAATGAAGATTCGCAACCGTC | |

**Supplementary Table 1.** Primers of qRT-PCR in verification of β-Actin and ten 2-DE identified protein genes (S and T correspond to protein spots of Zhongmu-1 and Jemalong A17).
