# Supplementary material for: Comparative Proteomic Analysis Reveals Differential Root Proteins in Medicago sativa and Medicago truncatula in Response to Salt Stress
Source: Front Plant Sci. 2016 Mar 31;7:424. doi: 10.3389/fpls.2016.00424 (PMC4814493; doi:10.3389/fpls.2016.00424)
Supplement: Supplementary Table 2 — Protein spot volume fold changes of all proteins identified in Zhongmu-1 and Jemalong A17. [file Table2.DOC]

**Supplementary Table 2.** The spot volume fold change of all the spots identified in Zhongmu-1 and Jamalong A17 root with significant abundance variation. Spot volume fold change = spot volume of 8 h / spot volume of control, “-” indicates the spots were only identified in sample of 8 h or control.

| **Spot ID** | **Spot volume of 0h**  **(%Vol)(Mean)** | **Spot volume of 8h**  **(%Vol)(Mean)** | **Spot volume fold change (Mean±SE)** |
| --- | --- | --- | --- |
| S1 | 0.00800 | 0.37968 | 47.46±2.51 |
| S2 | 0.01526 | 0.11395 | 7.47±0.86 |
| S3 | 0.00379 | 0.15396 | 40.62±3.26 |
| S4 | 0.00438 | 0.08209 | 18.74±1.58 |
| S5 | 0.00353 | 0.10532 | 29.84±2.45 |
| S6 | 0.00455 | 0.14589 | 32.06±2.57 |
| S7 | 0.00230 | 0.17452 | 75.88±5.36 |
| S8 | 0.00257 | 0.28037 | 109.09±5.16 |
| S9 | 0.00122 | 0.04851 | 39.76±1.23 |
| S10 | 0.00294 | 0.37834 | 128.69±3.36 |
| S11 | 0.00146 | 0.10630 | 72.81±5.28 |
| S12 | 0.05375 | 0.21956 | 4.08±0.27 |
| S13 | 0.00253 | 0.02741 | 10.83±0.85 |
| S14 | 0.00838 | 0.40250 | 48.03±1.29 |
| S15 | 0.00063 | 0.24368 | 386.79±8.63 |
| S16 | 0.00127 | 0.21487 | 169.19±6.59 |
| S17 | 0.05067 | 0.12949 | 2.56±0.35 |
| S18 | 0.00207 | 0.14476 | 69.93±4.56 |
| S19 | 0.00107 | 0.16169 | 151.11±6.89 |
| S20 | 0.07789 | 0.18140 | 2.33±0.35 |
| S21 | 0.00596 | 0.16572 | 27.81±2.55 |
| S22 | 0.00052 | 0.10387 | 199.75±8.95 |
| S23 | 0.08394 | 0.17728 | 2.118±0.38 |
| S24 | 0.00270 | 0.09506 | 35.21±5.36 |
| S25 | 0.00163 | 0.10325 | 63.34±4.58 |
| S26 | 0.07638 | 0.29982 | 3.93±0.56 |
| S27 | 0.06991 | 0.49643 | 7.10±0.89 |
| S28 | 0.00193 | 0.13981 | 72.44±5.12 |
| S29 | 0.12439 | 0.39658 | 3.19±0.31 |
| S30 | 0.02536 | 0.14920 | 5.88±0.52 |
| S31 | 0.07789 | 0.18140 | 2.33±0.13 |
| S32 | 0.52706 | 0.85106 | 1.61±0.17 |
| S33 | 0.45601 | 0.72635 | 1.59±0.11 |
| S34 | 0.00341 | 0.07818 | 22.93±3.66 |
| S35 | 0.00176 | 0.09539 | 54.20±8.59 |
| S36 | 0.00068 | 0.11537 | 169.66±7.25 |
| S37 | 0.00179 | 0.08169 | 45.64±4.56 |
| S38 | 0.00096 | 0.15688 | 163.42±6.95 |
| S39 | 0.00389 | 0.14673 | 37.72±7.33 |
| S40 | 0.00370 | 0.12342 | 33.36±2.59 |
| S41 | 0.00172 | 0.01523 | 8.85±0.93 |
| S42 | 0.00266 | 0.07166 | 26.94±4.26 |
| S43 | 0.00348 | 0.08113 | 23.31±2.38 |
| S44 | 0.01478 | 0.14661 | 9.92±0.56 |
| S45 | 0.01174 | 0.08083 | 6.89±0.15 |
| S46 | 0.00914 | 0.20605 | 22.54±3.26 |
| S47 | 0.00326 | 0.11529 | 35.37±2.51 |
| S48 | 0.00134 | 0.09221 | 68.81±4.23 |
| S49 | 0.00155 | 0.07255 | 46.81±3.98 |
| S50 | 0.02496 | 0.37698 | 15.10±1.25 |
| S51 | 0.00115 | 0.19218 | 167.11±5.62 |
| S52 | 0.00072 | 0.12565 | 174.51±6.95 |
| S53 | 0.00079 | 0.15761 | 199.51±15.89 |
| S54 | 0.06233 | 0.01159 | 0.19±0.03 |
| S55 | 0.14706 | 0.02622 | 0.18±0.05 |
| S56 | 0.12325 | 0.03030 | 0.25±0.04 |
| S57 | 0.22032 | 0.14853 | 0.54±0.10 |
| S58 | 0.23458 | 0.11523 | 0.49±0.05 |
| S59 | 0.13133 | 0.10287 | 0.63±0.03 |
| S60 | 0.78430 | 0.48027 | 0.61±0.08 |
| S61 | 0.25475 | 0.18769 | 0.54±0.06 |
| S62 | 0.06056 | 0.00529 | 0.09±0.02 |
| S63 | 0.15316 | 0.00334 | 0.02±0.01 |
| S64 | 0.15193 | 0.00572 | 0.04±0.01 |
| S65 | 0.09024 | 0.07143 | 0.46±0.02 |
| S66 | 0.13492 | 0.10142 | 0.61±0.06 |
| S67 | 0.22780 | 0.07812 | 0.34±0.02 |
| S68 | 0.28659 | 0.15840 | 0.55±0.08 |
| S69 | 0.10492 | 0.07638 | 0.73±0.07 |
| S70 | 0.03987 | 0.01166 | 0.29±0.05 |
| S71 | 0.07538 | 0.01693 | 0.22±0.06 |
| S72 | 0.43334 | 0.30452 | 0.62±0.04 |
| S73 | 0.29287 | 0.05974 | 0.20±0.02 |
| S74 | 0.56081 | 0.09200 | 0.16±0.01 |
| S75 | 0.07713 | - | - |
| S76 | 0.04784 | - | - |
| S77 | 0.12770 | 0.08504 | 0.59±0.06 |
| S78 | 0.20317 | 0.15240 | 0.55±0.05 |
| S79 | 0.65892 | 0.32567 | 0.49±0.08 |
| S80 | 0.05583 | - | - |
| S81 | 0.09330 | 0.06626 | 0.50±0.09 |
| S82 | 0.14026 | 0.07138 | 0.51±0.07 |
| S83 | 0.14876 | 0.06598 | 0.44±0.03 |
| S84 | 0.23767 | 0.15237 | 0.60±0.05 |
| S85 | 0.21932 | 0.12375 | 0.56±0.03 |
| S86 | 0.10872 | 0.05289 | 0.49±0.02 |
| S87 | 0.31119 | 0.23264 | 0.43±0.02 |
| S88 | 0.15344 | 0.06292 | 0.41±0.01 |
| S89 | 0.15625 | 0.06749 | 0.43±0.05 |
| S90 | 0.30005 | 0.22969 | 0.53±0.06 |
| S91 | 0.10408 | - | - |
| S92 | 0.13246 | 0.05640 | 0.43±0.04 |
| S93 | 0.33799 | 0.18872 | 0.56±0.08 |
| T1 | 0.01289 | 0.25607 | 19.87±1.25 |
| T2 | 0.00206 | 0.06212 | 30.16±3.26 |
| T3 | 0.01125 | 0.16376 | 14.56±0.78 |
| T4 | 0.09426 | 0.16292 | 1.73±0.15 |
| T5 | 0.25568 | 0.20018 | 1.57±0.11 |
| T6 | 0.18302 | 0.18214 | 1.54±0.03 |
| T7 | 0.10875 | 0.14011 | 1.56±0.08 |
| T8 | 0.09803 | 0.09517 | 1.99±0.07 |
| T9 | 0.30533 | 0.38341 | 1.58±0.14 |
| T10 | 0.13301 | 0.18054 | 2.11±0.06 |
| T11 | 0.21999 | 0.25960 | 1.63±0.05 |
| T12 | 0.41230 | 0.40143 | 1.56±0.09 |
| T13 | 0.03712 | 0.04923 | 2.40±0.07 |
| T14 | 0.01105 | 0.06028 | 5.46±0.15 |
| T15 | 0.12941 | 0.07156 | 1.60±0.05 |
| T16 | 0.06820 | 0.27945 | 4.10±0.79 |
| T17 | 0.00568 | 0.00946 | 1.67±0.15 |
| T18 | 0.00205 | 0.00752 | 3.67±0.63 |
| T19 | 0.00215 | 0.00821 | 3.82±0.45 |
| T20 | 0.00850 | 0.00763 | 2.07±0.15 |
| T21 | 0.00627 | 0.00723 | 2.75±0.16 |
| T22 | 0.06773 | 0.19358 | 2.86±0.25 |
| T23 | 0.16776 | 0.08542 | 0.51±0.05 |
| T24 | 0.19918 | 0.20499 | 0.53±0.06 |
| T25 | 0.18851 | 0.21481 | 0.61±0.04 |
| T26 | 0.11763 | 0.11348 | 0.62±0.04 |
| T27 | 0.14299 | 0.12523 | 0.60±0.03 |
| T28 | 0.18578 | 0.13098 | 0.44±0.09 |
| T29 | 0.20860 | 0.18108 | 0.53±0.07 |
| T30 | 0.22750 | 0.16673 | 0.56±0.03 |
