# Supplementary material for: Comparative Proteomic Analysis Reveals Differential Root Proteins in Medicago sativa and Medicago truncatula in Response to Salt Stress
Source: Front Plant Sci. 2016 Mar 31;7:424. doi: 10.3389/fpls.2016.00424 (PMC4814493; doi:10.3389/fpls.2016.00424)
Supplement: Supplementary Table 3 — Transcript expression analyses results of 10 differentially accumulated root proteins in Zhongmu-1 and Jemalong A17. [file Table3.DOC]

**Supplementary Table 3.** Transcript expression analyses results of 10 differentially accumulated root proteins in Zhongmu-1 and Jemalong A17.

| **Spot ID/gene** | **Relative expression level in Jemalong A17 (Mean±SE)** | | | | **Relative expression level in Zhongmu-1 (Mean±SE)** | | | |
| --- | --- | --- | --- | --- | --- | --- | --- | --- |
|  | 0h | 2h | 8h | 24h | 0h | 2h | 8h | 24h |
| Fructose-bisphosphate aldolase (S3/T1) | 1.00 | 2.13±0.25 | 10.3±0.63 | 9.55±0.46 | 1.00 | 5.51±0.36 | 15.63±0.42 | 18.71±0.41 |
| Heat shock protein (S28/T26) | 1.00 | 1.21±0.31 | 1.1±0.24 | 0.86±0.16 | 1.00 | 2.52±0.25 | 5.65±0.38 | 6.32±0.36 |
| TCP-1/cpn60 chaperonin family protein (S44/T2) | 1.00 | 1.58±0.19 | 2.83±0.35 | 1.97±0.24 | 1.00 | 1.84±0.20 | 2.67±0.24 | 4.24±0.24 |
| Cinnamyl alcohol dehydrogenase-like protein (S76) | 1.00 | 1.13±0.27 | 0.85±0.15 | 0.92±0.21 | 1.00 | 0.53±0.12 | 0.38±0.14 | 0.27±0.10 |
| Caffeoyl-CoA O-methyltransferase (S22/T10) | 1.00 | 2.25±0.23 | 3.89±0.34 | 4.56±0.35 | 1.00 | 4.25±0.27 | 8.63±0.29 | 11.27±0.35 |
| Phosphopyruvate hydratase (S53) | 1.00 | 1.89±0.15 | 2.35±0.20 | 2.06±0.217 | 1.00 | 5.89±0.63 | 13.15±0.47 | 8.54±0.72 |
| S-adenosyl-L-methionine synthase (S77) | 1.00 | 1.12±0.23 | 0.96±0.21 | 0.89±0.15 | 1.00 | 1.06±0.07 | 0.98±0.15 | 0.95±0.12 |
| CHP-rich zinc finger protein (T8) | 1.00 | 1.58±0.18 | 2.14±0.25 | 2.35±0.32 | 1.00 | 1.10±0.24 | 1.48±0.25 | 1.23±0.18 |
| Peroxidase (T20) | 1.00 | 1.21±0.21 | 1.87±0.20 | 1.69±0.17 | 1.00 | 1.42±0.18 | 3.56±0.46 | 8.21±0.38 |
| LEA14-like protein (T28) | 1.00 | 1.16±0.14 | 0.89±0.08 | 0.75±0.12 | 1.00 | 1.05±0.15 | 0.98±0.09 | 0.84±0.13 |
